# Extracellular Electron Uptake by Acetogenic Bacteria: Does H_2_ Consumption Favor the H_2_ Evolution Reaction on a Cathode or Metallic Iron?

**DOI:** 10.3389/fmicb.2019.02997

**Published:** 2020-01-10

**Authors:** Jo Philips

**Affiliations:** Department of Engineering, Aarhus University, Aarhus, Denmark

**Keywords:** acetogenesis, extracellular electron transfer mechanisms, energy conservation, ATP gain, Butler-Volmer equation, zero valent iron, biocathode, HER reaction

## Abstract

Some acetogenic bacteria are capable of using solid electron donors, such as a cathode or metallic iron [Fe(0)]. Acetogens using a cathode as electron donor are of interest for novel applications such as microbial electrosynthesis, while microorganisms using Fe(0) as electron donor cause detrimental microbial induced corrosion. The capacity to use solid electron donors strongly differs between acetogenic strains, which likely relates to their extracellular electron transfer (EET) mechanism. Different EET mechanisms have been proposed for acetogenic bacteria, including a direct mechanism and a H_2_ dependent indirect mechanism combined with extracellular hydrogenases catalyzing the H_2_ evolution reaction on the cathode or Fe(0) surface. Interestingly, low H_2_ partial pressures often prevail during acetogenesis with solid electron donors. Hence, an additional mechanism is here proposed: the maintenance of low H_2_ partial pressures by microbial H_2_ consumption, which thermodynamically favors the H_2_ evolution reaction on the cathode or Fe(0) surface. This work elaborates how the H_2_ partial pressure affects the H_2_ evolution onset potential and the H_2_ evolution rate on a cathode, as well as the free energy change of the anoxic corrosion reaction. In addition, the H_2_ consumption characteristics, i.e., H_2_ threshold (thermodynamic limit for H_2_ consumption) and H_2_ consumption kinetic parameters, of acetogenic bacteria are reviewed and evidence is discussed for strongly different H_2_ consumption characteristics. Different acetogenic strains are thus expected to maintain different H_2_ partial pressures on a cathode or Fe(0) surface, while those that maintain lower H_2_ partial pressures (lower H_2_ threshold, higher H_2_ affinity) more strongly increase the H_2_ evolution reaction. Consequently, I hypothesize that the different capacities of acetogenic bacteria to use solid electron donors are related to differences in their H_2_ consumption characteristics. The focus of this work is on acetogenic bacteria, but similar considerations are likely also relevant for other hydrogenotrophic microorganisms.

## Introduction

Acetogenic bacteria are a phylogenetically diverse group of microorganisms that share a unique metabolism for energy conservation and carbon fixation, i.e., the Wood–Ljungdahl pathway (Drake et al., 2008). This pathway reduces the electron acceptor CO_2_ with the electron donor H_2_ to acetyl-CoA for carbon fixation or to acetate or other organic compounds (e.g., ethanol) for energy conservation:

(Reaction 1) $$2{\text{CO}}_{2}+4{\text{H}}_{2}\to {\text{CH}}_{3}{\text{COO}}^{-}+{\text{H}}^{+}+2{\text{H}}_{2}\text{O}$$

$$\text{Δ}G^{0}{}_{acetogenesis}=-55.8\text{kJ}\cdot {\text{mol}}^{-1}$$

With *ΔG^0^_acetogenesis_* the Gibbs free energy change of acetogenesis in standard conditions (pH 0, all concentrations 1 M and all partial pressures 1 atm), in contrast to *ΔG^0^’_*acetogenesis*_* (−95.6 kJ⋅mol^–1^) in physiological standard conditions (pH 7).

Intriguingly, some acetogens can use solid electron donors, including a cathode (Nevin et al., 2010, 2011), metallic iron [Fe(0)] (Kato et al., 2015; Philips et al., 2019) and possibly reduced minerals. The use of a cathode as electron donor is of high interest for the development of innovative bioelectrochemical technologies. Microbial electrosynthesis, for instance, is a promising process for the conversion of excess renewable electricity and CO_2_ into biofuels or other valuable organic compounds using acetogenic bacteria as biocatalysts (Rabaey and Rozendal, 2010; Lovley and Nevin, 2013). In contrast, microorganisms using Fe(0) as electron donor cause microbial induced corrosion, resulting in severe damage to steel infrastructure (Enning and Garrelfs, 2014). Finally, the oxidation of reduced minerals by acetogens could have a still unknown impact on global biogeochemical cycles.

Not all tested acetogens are capable of using a cathode or Fe(0) as electron donor (Table 1). The highest electron uptake rates from cathodes have been reported for *Sporomusa ovata* strains (Nevin et al., 2010; Aryal et al., 2017). In contrast, the well-studied strain *Acetobacterium woodii* is not capable of withdrawing electrons from cathodes poised at a potential of −0.4 V vs. Standard Hydrogen Electrode (SHE) (potential slightly more positive than the standard potential for H_2_ evolution at pH 7, see calculations below). At more negative cathode potentials (< −0.6 V vs. SHE), almost all tested acetogenic strains withdraw cathodic electrons, with the exception of *Sporomusa aerivorans* (Table 1). Acetogenic communities enriched on cathodes (potentials usually ≤ −0.6 V vs. SHE) are often dominated by *Acetobacterium* species (Table 2). Only one study has performed a metagenome analysis to identify their cathode-dominating acetogen and found a *Acetobacterium wieringae* strain (Marshall et al., 2017). Interestingly, similar acetogenic genera are found in enrichments using Fe(0) as electron donor (Table 2) and acetogenic strains related to *Sporomusa sphaeroides* and *A. wieringae* have been isolated with Fe(0) (Kato et al., 2015; Philips et al., 2019; Table 1). Moreover, a limited to no Fe(0) corrosion enhancement was found for *A. woodii* (Table 1). Consequently, acetogenic strains likely use a similar mechanism to withdraw extracellular electrons from a cathode as from Fe(0), while not all acetogens have such a mechanism.

**TABLE 1:** Overview of acetogenic strains and their capacity to withdraw electrons from Fe(0) or a cathode.

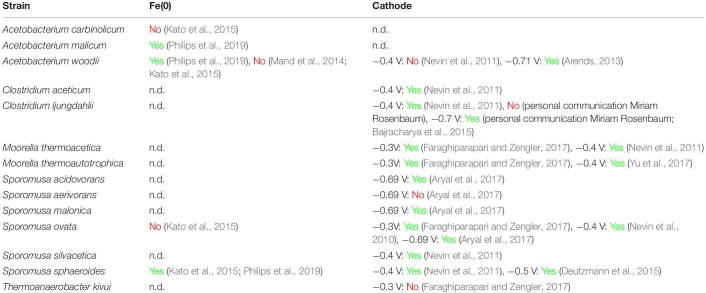

*The cathode potential (expressed vs. SHE) at which the electron uptake from a cathode was tested is indicated. n.d., not yet determined.*

**TABLE 2:** (Putative) acetogenic genera in acetogenic enrichments on Fe^0^ or cathodes.

**Solid electron donor**	**(Putative) acetogenic genera**	**References**
Fe(0)	*Acetobacterium*	Mand et al., 2014
Fe(0)	*Sporomusa, Clostridium*	Kato et al., 2015
Fe(0)	*Acetobacterium, Sporomusa, Clostridium, Acetoanaerobium*	Philips et al., 2019
Cathode (−0.6 V)	*Acetobacterium*	Marshall et al., 2012, 2013; LaBelle et al., 2014
Cathode (−0.7 V)	*Acetobacterium*	Su et al., 2013
Cathode (−0.85 V)	*Acetoanaerobium*	Jourdin et al., 2016
Cathode (−1.0 V)	*Acetobacterium*	Patil et al., 2015; Arends et al., 2017
Cathode (−1.0 V)	*Acetobacterium, Acetoanaerobium*	Xafenias and Mapelli, 2014
Cathode (−0.65 V)	*Acetobacterium*	Saheb-Alam et al., 2018

*Cathode potentials are expressed vs. SHE.*

This work first reviews the different extracellular electron transfer (EET) mechanisms that have been proposed for acetogenic bacteria. Next, an additional EET mechanism is proposed: the maintenance of low H_2_ partial pressures by H_2_ consumption, favoring H_2_ evolution by the cathode or Fe(0) surface. The H_2_ consumption characteristics of acetogens are further described using thermodynamic and kinetic calculations and a literature review. Finally, this work hypothesizes that the different capacities of acetogenic bacteria to use solid electron donors are related to differences in their H_2_ consumption characteristics. This work mainly focuses on acetogenic bacteria, but similar considerations are likely also valid for other hydrogenotrophic microorganisms.

## Extracellular Electron Transfer Mechanisms of Acetogens

**FIGURE 1:**
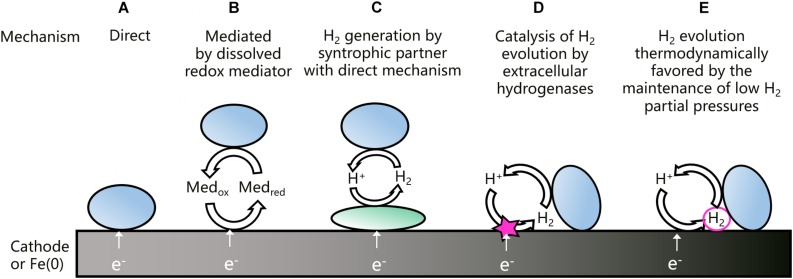
Schematic overview of the different EET mechanisms that have been proposed for the uptake of electrons from a cathode or Fe(0) by acetogenic bacteria (adjusted from Philips et al., 2019). Previously proposed EET mechanisms are direct **(A)**, mediated by dissolved redox mediators **(B)**, depended on H_2_ generation by a syntrophic partner with a direct mechanism **(C)**, or depended on H_2_ generation catalyzed by extracellular hydrogenases **(D)**. The last mechanism **(E)** is proposed and elaborated in this work.

An overview of the different EET mechanisms that have been proposed for acetogenic bacteria is shown in Figure 1. Direct EET (Figure 1A) is a mechanism that is well-studied in microorganisms using solid electron acceptors, as for instance *Geobacter* spp. (Philips et al., 2016). Other microorganisms, e.g., *Acidithiobacillus ferrooxidans*, use a direct EET mechanism to withdraw electrons from solid electron donors (Valdes et al., 2008). A direct EET mechanism typically involves outer-membrane bound cytochromes, transporting extracellular electrons from the inside to the outside of the cell or the other way around (Philips et al., 2016). A direct extracellular electron uptake has been proposed for acetogenic bacteria (Nevin et al., 2011; Kato et al., 2015), but clear evidence is still lacking. Moreover, *Moorella* and *Sporomusa* spp. have cytochromes, but most other acetogens have not (Moller et al., 1984; Schuchmann and Müller, 2014).

Some microorganisms excrete redox shuttles to mediate EET (Figure 1B). *Shewanella oneidensis* and *Pseudomonas aeruginosa*, for instance, use respectively flavins and phenazines to mediate the transport of electrons to an anode (Philips et al., 2016). Artificial mediators have been applied to improve the EET of acetogens from cathodes (Song et al., 2011), but acetogens were not found to excrete redox mediators to mediate EET from Fe(0) (Philips et al., 2019).

Another possibility is an indirect EET mechanism relying on the evolution of H_2_ on the cathode or Fe(0). All acetogens are capable of using H_2_ as electron donor. In addition, a cathode at a sufficiently low potential (calculated in detail below) generates H_2_ through proton reduction:

(Reaction 2) $$2{\text{H}}^{+}+2{\text{e}}^{-}\to {\text{H}}_{2}$$

Moreover, the presence of Fe(0) in anoxic conditions always leads to H_2_ generation through the anoxic corrosion reaction:

(Reaction 3) $$\text{Fe}\left(0\right)+2{\text{H}}^{+}↔{\text{Fe}}^{2+}+{\text{H}}_{2}$$

Nevertheless, an indirect EET mechanism depending on H_2_ has often been disregarded, because the acetate levels in biological treatments are often higher than can be explained by the H_2_ levels in abiotic controls. For instance, no H_2_ evolution was recorded for an abiotic cathode poised at −0.4 V vs. SHE, while several acetogenic strains consumed current and produced acetate at the same potential (Nevin et al., 2011). Similarly, some acetogens have a higher acetate production rate with Fe(0) as electron donor than can be explained just by chemically generated H_2_ (Kato et al., 2015; Philips et al., 2019). Consequently, an H_2_-dependent EET mechanism can only explain the extracellular electron uptake by acetogens, if the microorganisms somehow increase the H_2_ evolution on the cathode or Fe(0) surface.

Acetogens could increase H_2_ evolution on a cathode or Fe(0) through a syntrophic association with an electrotrophic microorganism, which produces H_2_ using a direct EET mechanism (Figure 1C). Such a mechanism was proposed for a cathodic microbial community dominated by *Acetobacterium* and *Desulfovibrio* spp. (Marshall et al., 2017). In addition, acetate production by *A. woodii* on a cathode poised at −0.4 V vs. SHE was facilitated through H_2_ generation by strain IS4 (Deutzmann and Spormann, 2017), i.e., a sulfate reducer isolated with Fe(0) as electron donor (Dinh et al., 2004) and possibly using cytochromes for a direct EET (Beese-Vasbender et al., 2015b).

Interestingly, some acetogenic strains can increase H_2_ evolution on a cathode or Fe(0), also without a syntrophic partner. Deutzmann et al. (2015) found that cell-free spent medium of *S. sphaeroides* increased the H_2_ evolution rate on a cathode poised at −0.5 V vs. SHE, while similar results were reported with Fe(0) for *Sporomusa* and *Acetobacterium* strains (Philips et al., 2019). Tremblay et al. (2019) detected H_2_ already at a cathode potential of −0.3 V vs. SHE with cell-free spent medium of *S. ovata*, while H_2_ could only be detected at −0.5 V vs. SHE in fresh medium. Deutzmann et al. (2015) suggested that spent medium contains extracellular enzymes, such as hydrogenases, that absorb on the cathode or Fe(0) surface and catalyze the H_2_ evolution reaction (Figure 1D). For the methanogen *Methanococcus maripaludis*, a heterodisulfide reductase supercomplex was isolated, which catalyzes the reduction of CO_2_ to formate at a cathode and Fe(0) surface (Lienemann et al., 2018). In addition, this methanogen excretes a [NiFe] hydrogenase to stimulate the anoxic corrosion reaction (Reaction 3) (Tsurumaru et al., 2018). So far, the H_2_ catalyzing components in the spent medium of acetogens have not yet been identified.

The recent evidence discussed above (Deutzmann et al., 2015; Philips et al., 2019; Tremblay et al., 2019), suggests that H_2_ plays an important role in the EET mechanism of acetogenic bacteria. Nevertheless, H_2_ can often not be detected during acetogenesis with a cathode or Fe(0) as electron donor (Jourdin et al., 2016; Philips et al., 2019). For that reason, this work proposes that the maintenance of low H_2_ partial pressures is an additional mechanism by which acetogens favor H_2_ evolution on a cathode or Fe(0) surface (Figure 1E). The importance of the H_2_ partial pressure for the H_2_ evolution reaction on a cathode or Fe(0) is elaborated next.

## Low H_2_ Partial Pressures Favor the H_2_ Evolution Reaction on a Cathode and Fe(0)

### Effect of the H_2_ Partial Pressure on Cathodic H_2_ Evolution

The cathode potential below which H_2_ evolution (Reaction 2) is thermodynamically favorable, i.e., the H_2_ evolution onset potential (*E*_*H^+ /H_2*_) (V), is given by the Nernst equation:

(1) $$E_{H^{+}/H_{2}}=E_{H^{+}/H_{2}}^{{}^{\circ }}-\frac{R\cdot T}{2\cdot F}\cdot l\,n\,\left(\frac{p_{H_{2}}}{{\left[H^{+}\right]}^{2}}\right)$$

With *R* the ideal gas constant (8.314 10^–3^ kJ⋅mol^–1^⋅K^–1^), *T* the temperature (K), *F* the Faraday constant (96.485 kJ⋅V^–1^) and *p*_*H_2*_ the H_2_ partial pressure (atm) and *[H^+^]* is the proton concentration (M). Equation 1 further neglects activity coefficients, assuming that activities can be approached by concentrations. The H_2_ partial pressure (atm) is used throughout this work even for conditions in solution (such as at the cathode or Fe(0) surface), but can be related to the dissolved H_2_ concentration using the Henri constant. *E*°_*H* + /*H*_2__ is the standard potential (pH 0, 1 atm H_2_) for H_2_ evolution, which is 0 V (i.e., the potential of the SHE), while *E*°’_*H^+ /H_2*_ is −0.414 V (pH 7, 1 atm H_2_).

**FIGURE 2:**
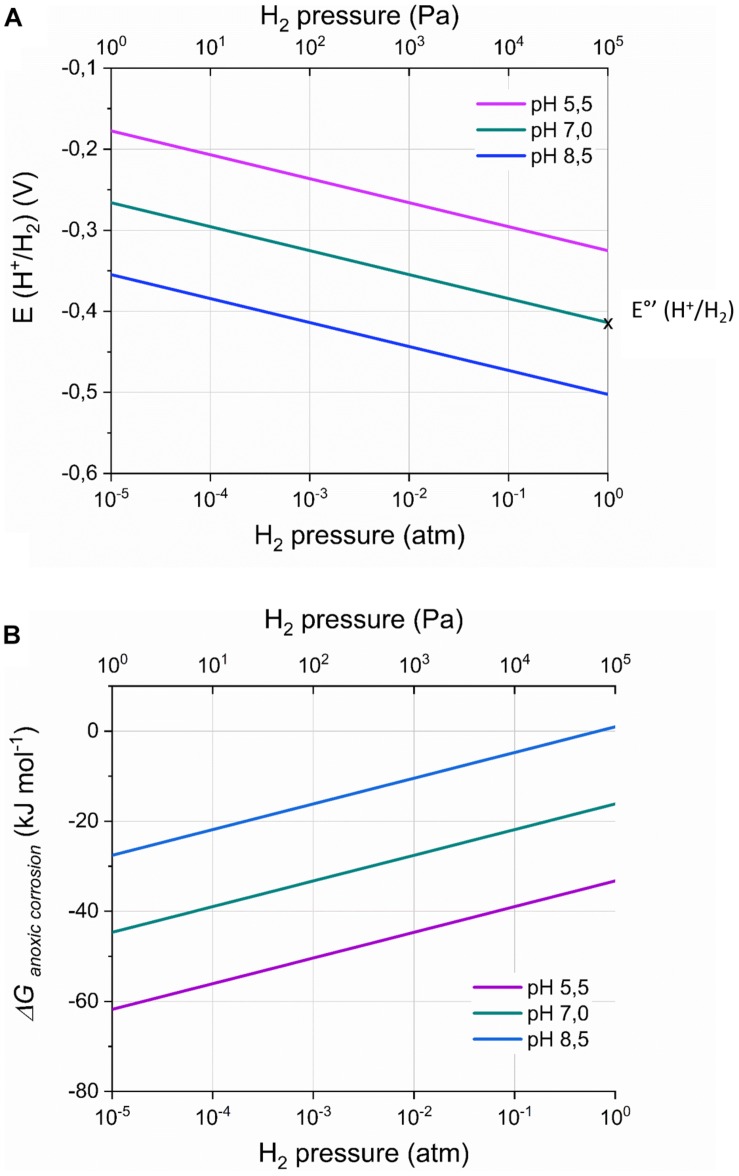
The onset potential for H_2_ evolution by a cathode *E(H^+^/H_2_)* (V) **(A)** and the Gibbs free energy change (*ΔG_*anoxic  corrosion*_*; kJ mol^– 1^) of the anoxic corrosion reaction **(B)** in function of the H_2_ partial pressure and the pH, according to respectively Equations 1 and 3. The standard H_2_ potential at pH 7 [*E*°*’(H^+^/H_2_*)] is indicated on the graph. *ΔG_*anoxic  corrosion*_* was calculated assuming a Fe^2+^ concentration of 1 mM. Remark the logarithmic scale for the H_2_ partial pressures.

Equation 1 demonstrates that the H_2_ evolution onset potential depends on the H_2_ partial pressure and the pH at the cathode surface (Figure 2A; Vincent et al., 2007; May et al., 2016). For instance, at a H_2_ partial pressure of 50 Pa (5 × 10^−4^ atm), the H_2_ evolution onset potential becomes – 0.316 V (pH 7), while at pH 5.5 [optimal pH for some acetogens (Liew et al., 2016)], the H_2_ evolution onset potential is – 0.325 V (1 atm H_2_). Consequently, H_2_ evolution can thermodynamically be favorable at cathode potentials less negative than −0.4 V vs. SHE, even though this potential is often used in bioelectrochemical studies to avoid H_2_ evolution.

The H_2_ evolution onset potential, and thus the H_2_ partial pressure (Equation 1), also affects the kinetics of the cathodic H_2_ evolution (Rheinlander et al., 2014), which can be described by the Butler–Volmer equation (Bard and Faulkner, 2001):

(2) $$j=j_{0}\cdot \left(e^{\frac{-\alpha \cdot 2\cdot F}{R\cdot T}\cdot \left(E_{e\,l\,e\,c\,t\,r\,o\,d\,e}-E_{H^{+}/H_{2}}\right)}-e^{\frac{\left(1-\alpha \right)\cdot 2\cdot F}{R\cdot T}\cdot \left(E_{e\,l\,e\,c\,t\,r\,o\,d\,e}-E_{H^{+}/H_{2}}\right)}\right)$$

With *j* the current density (A⋅cm^–2^), *j*_0_ the exchange current density (A⋅cm^–2^), *α* the transfer coefficient (-) (usually approximated by 0.5) (Bard and Faulkner, 2001) and *E*_*electrode*_ the potential at which the electrode is poised. The left exponential expresses the cathodic reaction (H^+^ to H_2_ reduction), while the right exponential expresses the anodic reaction (H_2_ to H^+^ oxidation). When *E*_*electrode*_ is *E*_*H^+ /H_2*_, (thermodynamic equilibrium) both the anodic and the cathodic current density become *j*_0_, hence the net current density is zero. Remark that Equation 2 is only valid if mass transfer is not limiting, e.g., when the concentrations at the electrode surface are the same as in the bulk liquid, which is only true at electrode potentials close to *E*_*H^+ /H_2*_ or, in other words, at very low currents.

**FIGURE 3:**
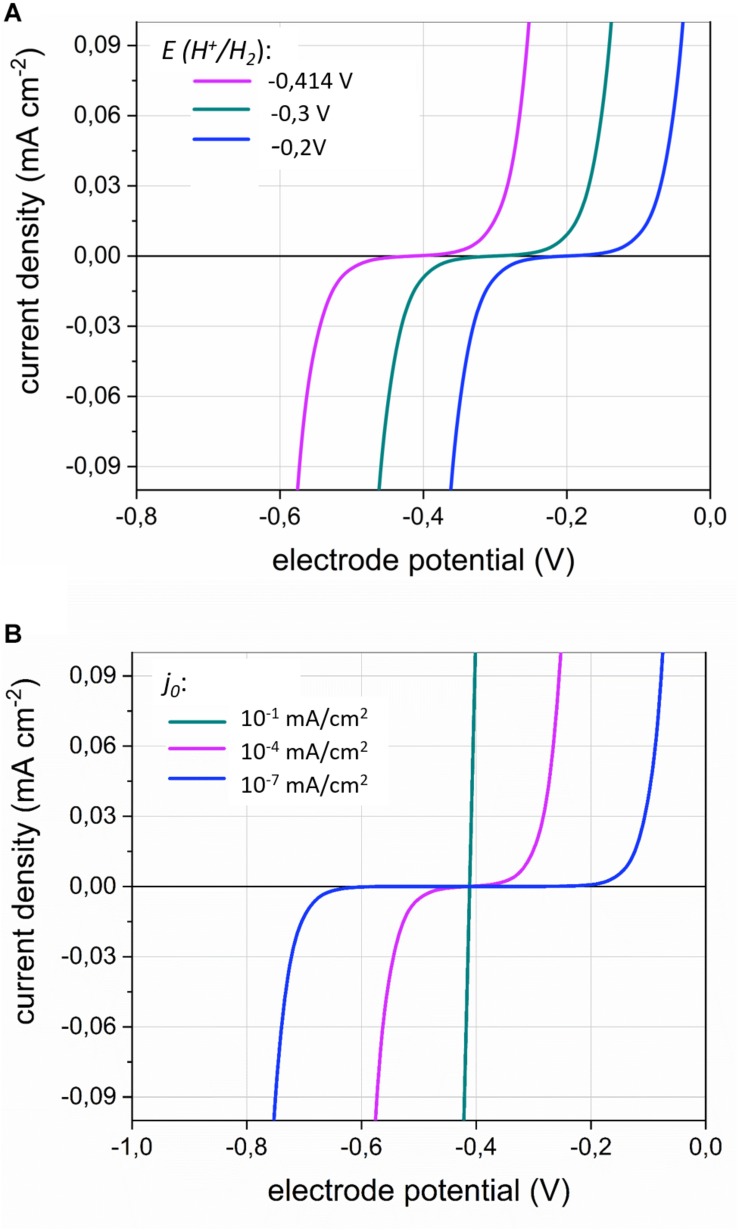
The current density *j* (mA⋅cm^–2^) in function of the electrode potential (V) according to the Butler–Volmer Equation (Equation 2) for **(A)** different values of the H_2_ evolution onset potential *E*(*H*^+^/*H*_2_); (with *j*_0_ = 1⋅10^–7^ mA⋅cm^–2^) and **(B)** different values of the exchange current density *j*_0_ (with *E*(*H*^+^/*H*_2_) = −0.414V). The transfer coefficient α was set to 0.5. The inflection point of the curves represents *E(H^+^/H_2_)*.

The importance of the H_2_ evolution onset potential for the current density (equivalent to the H_2_ evolution rate) is illustrated in Figure 3A. A less negative *E*_*H^+ /H_2*_ value, for instance due to a lower H_2_ partial pressure, allows cathodic current at less negative potentials.

In addition, the effect the exchange current density *j*_0_ is illustrated in Figure 3B. The exchange current density inversely relates to the activation overpotential (Bard and Faulkner, 2001), as a smaller exchange current density entails that a more negative electrode potential is needed to enable a substantial cathodic current (Figure 3B). In bioelectrochemical studies, the overpotentials for cathodic H_2_ evolution are often high (0.2 V more negative than *E*°*’_*H^+ /H_2*_*), due to the low reactivity of the often used carbon-based electrode materials. Choosing for more reactive cathode materials (materials with high *j*_0_) strongly reduces the activation overpotential of the H_2_ evolution reaction (Jeremiasse, 2011). Such materials facilitate the cathodic electron uptake by acetogens at less negative cathode potentials than are required with unreactive electrode materials (Kracke et al., 2019; Tian et al., 2019). Moreover, cell-free spent medium of *S. ovata* was also found to decrease the overpotential for cathodic H_2_ evolution (Tremblay et al., 2019), likely because it contains hydrogenase enzymes or other components catalyzing the H_2_ evolution (Figure 1D).

In summary, Equation 2 demonstrates that the current density (H_2_ evolution rate) depends both on the H_2_ evolution onset potential (and thus the H_2_ partial pressure; thermodynamic effect) and on the exchange current density (kinetic effect, related to the electrode material and catalysis by enzymes), when mass transfer is not limiting.

### Effect of the H_2_ Partial Pressure on Anoxic Fe(0) Corrosion

The H_2_ partial pressure also affects the anoxic chemical corrosion reaction, as the Gibbs free energy change of Reaction 3 (*ΔG_*anoxic  corrosion*_*) depends on the H_2_ partial pressure and pH at the Fe(0) surface (Figure 2B):

$$\Delta \,G_{a\,n\,o\,x\,i\,c\,c\,o\,r\,r\,o\,s\,i\,o\,n}=\Delta \,G_{a\,n\,o\,x\,i\,c\,c\,o\,r\,r\,o\,s\,i\,o\,n}^{{}^{\circ }}$$

(3) $$+R\cdot T\cdot l\,n\,\left(\frac{p_{H_{2}}\cdot \left[F\,e^{2+}\right]}{{\left[H^{+}\right]}^{2}}\right)$$

With *[Fe^2+^]* the dissolved Fe^2+^ concentration (M) and *ΔG*°*_*anoxic  corrosion*_* the standard Gibbs free energy change (−78.9 kJ⋅mol^–1^, pH 0). Consequently, hydrogenotrophic microorganisms can thermodynamically favor H_2_ evolution on Fe(0) by maintaining low H_2_ partial pressures on the Fe(0) surface. For instance, *A. woodii* maintained a H_2_ partial pressure on Fe(0) of 150 Pa (1.5 × 10^−3^ atm) (Philips et al., 2019), leading to *ΔG_*anoxic  corrosion*_* of −32 kJ⋅mol^–1^, while this is only −22 kJ⋅mol^–1^ in abiotic conditions (0.1 atm or 10.000 Pa H_2_; assuming *[Fe^2+^]* of 1 mM) (Philips et al., 2019). All other strains tested in the same study maintained lower H_2_ partial pressures on Fe(0) than *A. woodii* [below the detection limit of a TCD detector (40 Pa)] (Philips et al., 2019), thus leading to a *ΔG_*anoxic  corrosion*_* value at least as negative as −36 kJ⋅mol^–1^.

Also the rate of the anoxic chemical corrosion reaction likely depends on the H_2_ partial pressure. In addition, Reaction 3 was found to be catalyzed by hydrogenase enzymes (Bryant and Laishley, 1990; Da Silva et al., 2004; Rouvre and Basseguy, 2016). Consequently, the rate of the anoxic corrosion reaction likely depends both on the H_2_ partial pressure (thermodynamic effect) and on enzymatic catalysis (kinetic effect), similar as for a cathode.

In general, the H_2_ partial pressure at the cathode or Fe(0) surface results from the balance (steady-state) between the H_2_ evolution rate on the cathode or Fe(0) and the H_2_ consumption rate by the microorganisms. For that reason, the H_2_ consumption characteristics of acetogenic bacteria are discussed next.

## H_2_ Consumption Characteristics of Acetogenic Bacteria

The consumption of H_2_ by any microorganism is described by its H_2_ threshold (the thermodynamic limit of H_2_ consumption) and its H_2_ consumption kinetics. Below, the theoretical H_2_ threshold is calculated for acetogens and experimentally determined values for the H_2_ threshold and H_2_ consumption kinetic parameters of acetogens are reviewed.

### The Theoretical H_2_ Threshold of Acetogens

Microorganisms do not completely consume their substrates due to bioenergetic constraints. The theoretical limit for H_2_ consumption by acetogenic bacteria is the H_2_ partial pressure at which Reaction 1 reaches its thermodynamic equilibrium:

$$\Delta \,G_{a\,c\,e\,t\,o\,g\,e\,n\,e\,s\,i\,s}=\Delta \,G_{a\,c\,e\,t\,o\,g\,e\,n\,e\,s\,i\,s}^{0}$$

(4) $$+R\cdot T\cdot l\,n\,\left(\frac{\left[C\,H_{3}\,C\,O\,O^{-}\right]\cdot \left[H^{+}\right]}{p_{C\,O_{2}}^{2}\cdot p_{H_{2}}^{4}}\right)=0$$

With *[CH_3_COO^–^]* and *[H^+^]* respectively the acetate and proton concentrations (M) and *p*_*CO_2*_ and *p*_*H_2*_ respectively the CO_2_ and H_2_ partial pressures (atm). The minimum H_2_ partial pressure at which a reaction is thermodynamically feasible is called the H_2_ threshold (*θ_*H_2*_*; atm) (Cord-Ruwisch et al., 1988) and can for acetogens be derived as:

(5) $$\theta_{H_{2}}=e^{\left(\frac{1}{4}\cdot \left(\frac{\Delta \,G_{a\,c\,e\,t\,o\,g\,e\,n\,e\,s\,i\,s}^{{}^{\circ }}}{R\cdot T}+l\,n\,\left(\frac{\left[C\,H_{3}\,C\,O\,O^{-}\right]\cdot \left[H^{+}\right]}{p_{C\,O_{2}}^{2}}\right)\right)\right)}$$

Using pH 7, a temperature of 298 K, a CO_2_ partial pressure of 0.2 atm and an acetate concentration of 2 mM [i.e., relevant physiological conditions for acetogens using a cathode or Fe(0) as electron donor (Nevin et al., 2010; Kato et al., 2015; Aryal et al., 2017; Philips et al., 2019)], the H_2_ threshold becomes 3⋅10^–5^ atm or 3 Pa (30 ppm or 0.003%, assuming 1 atm total pressure). Experimental H_2_ thresholds for acetogens are always higher than this value (discussed below). This is likely because Reaction 1 does not account for the coupling of acetogenesis to energy conservation. Indeed, calculations of the Gibbs free energy change at experimentally derived H_2_ thresholds found a critical Gibbs free energy change, which was not zero but slightly negative (Seitz et al., 1990). This critical Gibbs free energy change likely reflects the energy needed for the microbial metabolism. Accordingly, Reaction 1 should be written as (Poehlein et al., 2012):

$$2{\text{CO}}_{2}+4{\text{H}}_{2}+n\text{ADP}+n{\text{P}}_{i}$$

Reaction 4 $$\to {\text{CH}}_{3}{\text{COO}}^{-}+{\text{H}}^{+}+n\text{ATP}+2{\text{H}}_{2}\text{O}$$

With *n* the number of ATP molecules gained per molecule of acetate (i.e., the ATP gain). Consequently, the expression for the H_2_ threshold becomes:

(6) $$\theta_{H_{2}}=e^{\left(\frac{1}{4}\cdot \left(\frac{\Delta \,G_{a\,c\,e\,t\,o\,g\,e\,n\,e\,s\,i\,s}^{{}^{\circ }}+n\cdot \Delta \,G_{A\,D\,P/A\,T\,P}}{R\cdot T}+l\,n\,\left(\frac{\left[C\,H_{3}\,C\,O\,O^{-}\right]\cdot \left[H^{+}\right]}{p_{C\,O_{2}}^{2}}\right)\right)\right)}$$

with *ΔG_*ADP/ATP*_* the Gibbs free energy change for the phosphorylation of ADP to ATP in physiological conditions (also called the phosphorylation potential). Reported values for *ΔG_*ADP/ATP*_* range between 30 and 80 kJ⋅mol^–1^ (Thauer et al., 1977; Atkins and De Paula, 2011). For *A. woodii*, a phosphorylation potential of only 32 kJ⋅mol^–1^ has been measured (Spahn et al., 2015), but for other acetogens this value is unknown. For the calculation of the H_2_ threshold of different acetogens described here, a value for *ΔG*°*_*ADP/ATP*_* of 50 kJ⋅mol^–1^ (Poehlein et al., 2012) was used, in order not to underestimate the H_2_ threshold.

Equation 6 shows that the H_2_ threshold depends on the ATP gain *n*, which is maximally 1.9 (*ΔG^0^’_*acetogenesis*_* divided by *ΔG_*ADP/ATP*_*), but depends on the energy conservation mechanism of the acetogenic strain (Schuchmann and Müller, 2014).

**FIGURE 4:**
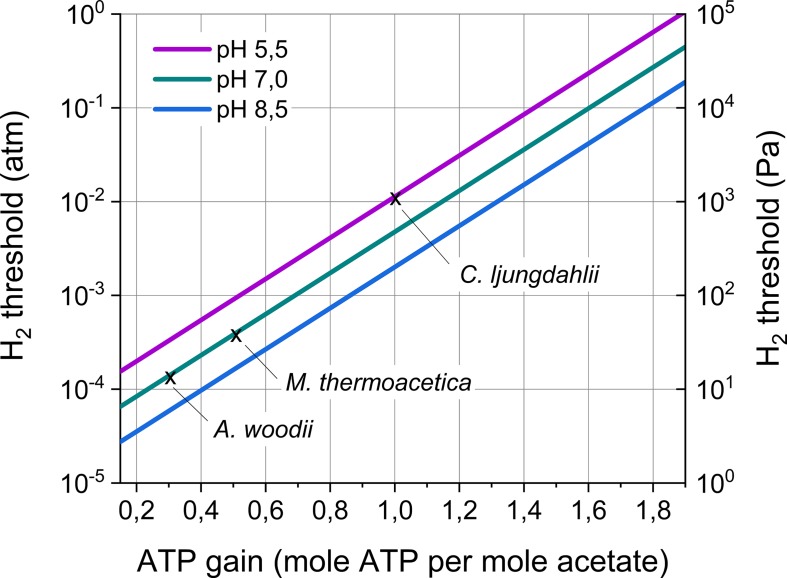
H_2_ threshold in function of the ATP gain of acetogenesis and the pH. The H_2_ threshold was calculated according to Equation 6 assuming an acetate concentration of 2 mM and a CO_2_ partial pressure of 0.2 atm. Temperature effects were not included in this graph. The H_2_ thresholds for three model acetogens (as in Table 3) are indicated. Remark the logarithmic scale for the H_2_ threshold.

All acetogenic bacteria share the Wood–Ljungdahl pathway, as the enzymes forming this pathway are highly conserved among acetogens (Schuchmann and Müller, 2014). However, the carbon flow through the Wood–Ljungdahl pathway does not lead to energy conservation. Acetogens conserve energy using chemiosmotic ion gradient-driven phosphorylation. The cation generating this gradient (Na^+^ or H^+^), the energy-conserving module (Rnf or Ech complex), as well as other components (electron bifurcating enzymes) creating the electron flow, differ between acetogenic bacteria (Schuchmann and Müller, 2014). So far, only for few model acetogenic strains the energy conservation machinery is (almost) fully unraveled and the theoretical ATP gain *n* has become available (Table 3). This ATP gain ranges between 0.3 for *A. woodii* to 1 for *Clostridium autoethanogenum*. Based on Equation 6, this entails that the H_2_ thresholds for these model strains range from 14 to 1160 Pa (including different optimal temperatures and pH) (Table 3). Moreover, based on recently sequenced genomes, it is plausible that a wide variability in the energy conservation mechanism of acetogenic strains exists (Poehlein et al., 2016). In theory, the ATP gain *n* could range from 0.15 (Mock et al., 2015) to maximally 1.9, entailing H_2_ thresholds ranging over five order of magnitudes (Figure 4). Consequently, acetogenic bacteria strongly differ in the lowest H_2_ partial pressure they can use. Strains with a high H_2_ threshold (high ATP gain) obtain high energy by performing acetogenesis, but cannot grow at low H_2_ partial pressures. In contrast, strains with a low H_2_ threshold (low ATP gain) gain low energy from acetogenesis, but have the advantage of being able to grow at low H_2_ partial pressures.

**TABLE 3:** Comparison of theoretical H_2_ thresholds of three model acetogens.

**Strain**	***Acetobacterium woodii***	***Clostridium autoethanogenum***	***Moorella thermoacetica***
Temperature optimum (°C)	25	37	55
pH optimum	7.0	5.5	7.0
ATP gain *n* (mole ATP/mole acetate)^a^	0.3^b^	1^c^	0.5^d^
**Hydrogen threshold (Pa)^e^**	**14**	**1160**	**51**

*^*a*^Only ATP gains for the formation of acetate are considered here, ATP gains for the formation of other products are different (Bertsch and Müller, 2015; Mock et al., 2015). ^*b*^The energy conservation mechanism of *A. woodii* is completely unraveled and the exact ATP gain known (Schuchmann and Müller, 2014). ^*c*^This ATP gain is an assumed value (Mock et al., 2015), as the energy conservation mechanism of *C. autoethanogenum* is not yet completely unraveled. ^*d*^This ATP gain is an assumed value (Schuchmann and Müller, 2014; Basen and Müller, 2017), as the energy conservation mechanism of *M. thermoacetica* is not yet completely unraveled. ^*e*^Calculated using Equation 6 and the ATP gain *n* and the pH and temperature given in the table and assuming an acetate concentration of 2 mM and CO_2_ partial pressure of 0.2 atm. The effect of the temperature on *ΔG^0^_*acetogenesis*_* and *ΔG_*ADP/ATP*_* was neglected, as the temperature effect on *ΔG_*ADP/ATP*_* is not known.*

Previously, differences in the H_2_ threshold of methanogens were similarly linked to different ATP gains (Thauer et al., 2008). The above analysis, however, only holds as long as the metabolism is coupled to energy generation, as it does not incorporate the possibility that acetogenesis continues decoupled from energy generation (Schuchmann and Müller, 2014). Moreover, the above reactions do not incorporate the consumption of H_2_ and CO_2_ for biomass formation. Furthermore, different energy conservation strategies could exist in a single strain (Mock et al., 2015) and be expressed depending on the H_2_ partial pressure. Consequently, further investigations of the energy conservation mechanisms of acetogenic bacteria will be highly important to better understand their H_2_ threshold.

### Experimental H_2_ Thresholds of Acetogens

Experimental H_2_ thresholds have been reported for several acetogenic strains (Table 4). These values are usually measured as the constant H_2_ partial pressure that remains after H_2_ depletion (other nutrients not limiting) (Cord-Ruwisch et al., 1988; Poehlein et al., 2012). Reported H_2_ thresholds range over two orders of magnitudes (Table 4), but strong value variability was reported even for the same strain, as experimental H_2_ thresholds for *A. woodii* for instance range from 14 to 250 Pa (Table 4). This variability is likely due to varying experimental conditions, as the H_2_ threshold depends in theory on the CO_2_ partial pressure, the acetate concentration, the pH, the total pressure and the temperature (Equations 5 and 6). Conrad and Wetter (1990) and Kotsyurbenko et al. (2001) nicely demonstrated that experimental H_2_ thresholds followed the theoretical temperature dependence (Equation 5), as long as the temperature remained in the strain’s optimal temperature range. The effect of the other parameters on the experimental H_2_ thresholds has much less been studied and often these parameters are not reported together with the experimental H_2_ threshold values. Fortunately, some studies have used the same experimental conditions to determine the H_2_ threshold of different acetogenic strains and demonstrated significant differences in experimental H_2_ thresholds between strains (Cord-Ruwisch et al., 1988; Leclerc et al., 1997; Le Van et al., 1998).

**TABLE 4:** Experimental H_2_ thresholds for different acetogens performing acetogenesis from CO_2_ and H_2_.

**Strain**	**H_2_ threshold (Pa)**	**Conditions^a^**	**References**
*Acetitomaculum ruminis*	384^b^	38°C, 24% CO_2_	Le Van et al., 1998
*Acetobacterium bakii*	8–80	4–30°C, 20% CO_2_	Kotsyurbenko et al., 2001
*Acetobacterium carbinolicum*	96^b^	28–34°C, 20% CO_2_, 1 bar	Cord-Ruwisch et al., 1988
	5–20	5–25°C, 2 mM acetate, pH 7, 28 kPa CO_2_	Conrad and Wetter, 1990
*Acetobacterium fimetarium*	15–80	4–30°C, 20% CO_2_	Kotsyurbenko et al., 2001
*Acetobacterium paludosum*	15–150	4–30°C, 20% CO_2_	Kotsyurbenko et al., 2001
*Acetobacterium psammolithicum*	53^c^	30°C	Krumholz et al., 1999
*Acetobacterium tundrae*	10–100	4–30°C, 20% CO_2_	Kotsyurbenko et al., 2001
*Acetobacterium woodii*	53^b^	28–34°C, 20% CO_2_, 1 bar	Cord-Ruwisch et al., 1988
	250	30°C, 20% CO_2_	Poehlein et al., 2012
	14–55	15–30°C, 2 mM acetate, pH 7, 28 kPa CO_2_	Conrad and Wetter, 1990
	37^b^	30°C, 24% CO_2_	Le Van et al., 1998
	18^b^	30°C, 20% CO_2_, 1 atm	Leclerc et al., 1997
*Moorella thermoacetica*	156^b^	30°C, 20% CO_2_, 1 atm	Leclerc et al., 1997
*Sporomusa termitida*	84^b^	28–34°C, 20% CO_2_, 1 bar	Cord-Ruwisch et al., 1988
	88^b^	30°C, 24% CO_2_	Le Van et al., 1998
*Thermoanaerobacter kivui*	300–600	50–60°C, 2 mM acetate, pH 7, 28 kPa CO_2_	Conrad and Wetter, 1990
*Treponema primitia*	50^b^	30°C, 20% CO_2_	Graber and Breznak, 2004

*^*a*^Conditions are mentioned as far as they are reported in the cited reference. ^*b*^H_2_ thresholds were converted from ppm values assuming a total pressure of 1 atm. ^*c*^The H_2_ threshold was converted using a Henri coefficient for H_2_ of 7.78 10^–3^ mol m^–3^ kPa^–1^.*

This work advocates the reporting of experimental H_2_ thresholds (as well as of the experimental conditions of the measurements) of H_2_ consuming anaerobic microorganisms, as this parameter can easily be determined and forms a highly valuable measure to assess bioenergetics, and possibly also the energy conservation mechanism, of new and already-known strains.

### H_2_ Consumption Kinetics of Acetogens

**FIGURE 5:**
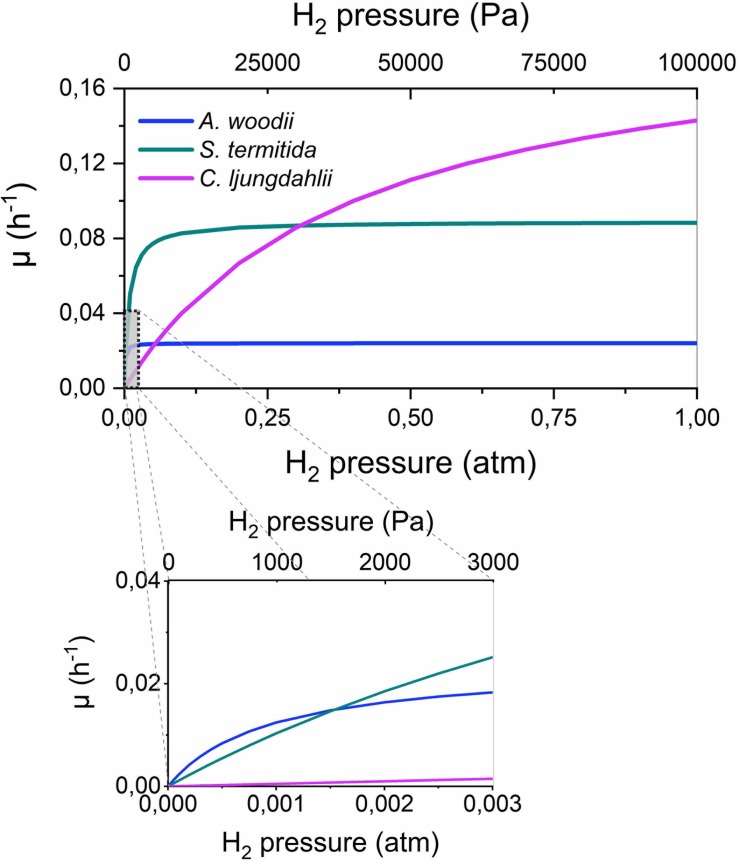
The cell specific growth rate (μ, h^– 1^) in function of the H_2_ partial pressure for three acetogens, according to Equation 7 and the kinetic parameters of Table 5. The H_2_ threshold was not incorporated in this graph.

Very limited information on the H_2_ consumption kinetics of acetogenic bacteria is available in literature, except for frequently reported doubling times (overview in Bengelsdorf et al., 2018). The kinetic parameters for H_2_ consumption were previously reported only for four acetogenic strains (Table 5). These strains strongly differ in their H_2_ consumption kinetics, as the maximum cell specific growth rate (μ*_*max*_*) differs one order of magnitude between these strains, while the Monod or half saturation constant (*K*_*H_2*_), i.e., a measure for the affinity of the strains for H_2_, ranges over more than two orders of magnitude. The importance of these different kinetic parameters becomes clear in Figure 5, plotting the cell specific growth rate (μ) in function of the H_2_ partial pressure according to the Monod Equation:

(7) $$\mu =\frac{\mu_{m\,a\,x}\cdot p_{H_{2}}}{K_{H_{2}}+p_{H_{2}}}$$

**TABLE 5:** Monod kinetic parameters for H_2_ consumption by acetogenic strains with *K*_*H_2*_ (Pa) the Monod or half saturation constant (i.e., a measure for the affinity of the strains for H_2_) and μ*_*max*_* the maximum cell specific growth rate (h^–1^).

**Strain**	***K*_*H_2*_ (Pa)**	**μ*_*max*_* (h^–1^)**	**Temperature (°C)**	**References**
*Acetobacterium woodii*	94	0.024	30	Peters et al., 1998
*Acetobacterium bakii*	520	n.d.^a^	30	Kotsyurbenko et al., 2001
*Sporomusa termitida*	770^b^	0.09^c^	30	Breznak et al., 1988
*Clostridium ljungdahlii*	42000	0.195	37	Mohammadi et al., 2014

*^*a*^A value of 760 nmol⋅h^–1^ was reported by Kotsyurbenko et al. (2001), but could not be converted to μ*_*max*_*, as no growth yield was reported. ^*b*^Converted using a Henri coefficient for H_2_ of 7.78 10^–3^ mol m^–3^ kPa^–1^. ^*c*^Converted from the doubling time *g* (h) using μ*_*max*_* = ln 2⋅*g*^–1^.*

This figure shows that strains with a high μ*_*max*_*, such as *C. ljungdahlii*, have the highest growth rate and thus a competitive advantage at high H_2_ partial pressures (> 0.25 atm). In contrast, at intermediate H_2_ partial pressures, strains with an intermediate *K*_*H_2*_ value, such as *S. termitida*, have a competitive advantage, while at very low H_2_ partial pressures (< 0.0015 atm), strains with a strong affinity for H_2_ (low *K*_*H_2*_), such as *A. woodii*, have the highest growth rate. Possibly, these differences explain why acetogenic *Clostridium* spp. are well suited for gas fermentations (Liew et al., 2016), while *Acetobacterium* and *Sporomusa* species are often found on cathodes or Fe(0), where low H_2_ partial pressures prevail (Table 2). Importantly, Figure 5 also shows that the highest growth rates are only obtained at high H_2_ partial pressures, possibly impeding the production rates attainable with microbial electrosynthesis in comparison to gas fermentation.

Equation 7 can further be extended to also include the H_2_ threshold (Kotsyurbenko et al., 2001):

(8) $$\mu =\frac{\mu_{m\,a\,x}\cdot \left(p_{H_{2}}-\theta_{H_{2}}\right)}{K_{H_{2}}+\left(p_{H_{2}}-\theta_{H_{2}}\right)}$$

This equation was not used for Figure 5, as for none of the strains, all three parameters (μ*_*max*_*, *K*_*H_2*_ and *θ_*H_2*_*) are reported.

## Discussion

Several acetogenic bacteria are capable of using solid electron donors (Tables 1, 2), implying that these strains have an extracellular electron uptake mechanism. Different EET mechanisms have been proposed for acetogenic bacteria (Figure 1). Recent evidence suggests that an H_2_-dependent indirect EET mechanism is combined with a mechanism to increase the H_2_ evolution reaction rate on the cathode or Fe(0) surface (Deutzmann et al., 2015; Jourdin et al., 2016; Tremblay et al., 2019). In addition, low H_2_ partial pressures often prevail during acetogenesis with a solid electron donor (< 50 Pa) (Nevin et al., 2010; Deutzmann et al., 2015; Jourdin et al., 2016; Philips et al., 2019). This work explained that the H_2_ partial pressure affects the H_2_ evolution onset potential (Equation 1 and Figure 2A) and the H_2_ evolution rate (current) (Equation 2 and Figure 3A) at a cathode, as well the Gibbs free energy change of the anoxic corrosion reaction (Equation 3 and Figure 2B) and likely also the rate of the corrosion reaction. Consequently, hydrogenotrophic microorganisms could favor the H_2_ evolution reaction by maintaining low H_2_ partial pressures at the cathode or Fe(0) surface (Figure 1E).

The steady-state H_2_ partial pressure at the material surface results from the balance between the H_2_ evolution reaction and microbial H_2_ consumption. Here, the H_2_ consumption characteristics of acetogenic bacteria were reviewed, which suggested that acetogens differ in their H_2_ threshold (thermodynamic limit for H_2_ consumption) (Figure 4 and Tables 3, 4) and their H_2_ consumption kinetics (Figure 5 and Table 5). This entails that different acetogens likely maintain different H_2_ partial pressures on the surface of a cathode or Fe(0). Therefore, I hypothesize that acetogens that maintain lower H_2_ pressures (strains with a lower H_2_ threshold and/or higher H_2_ affinity) more strongly increase the H_2_ evolution reaction on a cathode or Fe(0). Consequently, the differences in the capacities of acetogenic bacteria to use solid electron donors (Table 1) could be related to differences in their H_2_ consumption characteristics.

The lowest theoretical H_2_ threshold and highest H_2_ affinity (lowest *K*_*H_2*_) was reported for *A. woodii* (Tables 3, 5). This contradicts with my hypothesis, as *A. woodii* is not capable of withdrawing cathodic electrons at a cathode potential of −0.4 V vs. SHE and does not increase Fe(0) corrosion or just to a limited extent (Table 1). However, information on the H_2_ consumption characteristics of acetogens is limited to just a few strains (Tables 3–5), not including the acetogenic strains most capable of using a cathode or Fe(0) as electron donor, i.e., *S. ovata*, *S. sphaeroides*, and *A. wieringae* (Kato et al., 2015; Aryal et al., 2017; Marshall et al., 2017; Philips et al., 2019). These and other acetogenic strains could have a lower H_2_ threshold and/or a lower *K*_*H_2*_ than *A. woodii* (Figure 4). *A. woodii* maintained a H_2_ partial pressure on Fe(0) of 150 Pa, while all other strains tested in the same study maintained H_2_ partial pressures on Fe(0) below the detection limit (40 Pa) (Philips et al., 2019), indicating that the other strains have better H_2_ consumption characteristics to maintain low H_2_ partial pressures on the Fe(0) surface than *A. woodii*. Experimental studies are needed to determine the H_2_ consumption characteristics (H_2_ threshold and kinetic parameters) of more acetogenic strains and to investigate if those H_2_ consumption characteristics relate to the capacity of acetogens to use solid electron donors.

It should be noted that H_2_ consumption depends on more factors than just the H_2_ threshold and the H_2_ consumption kinetic parameters. The number of cells on the surface also affects the H_2_ consumption rate, thus attachment and biofilm formation properties are important. In addition, several components, such as dissolved Fe(II), and a pH deviating from the optimal pH could inhibit H_2_ consumption. Future studies assessing also these factors will be important to fundamentally understand the role of H_2_ consumption in increasing the H_2_ evolution reaction.

This work suggests that microbial H_2_ consumption favors cathodic H_2_ evolution. Interestingly, the increase of the anoxic corrosion reaction by microbial H_2_ scavenging is a well-known theory, often referred to as “cathodic depolarization”, initially proposed in 1934 (von Wolzogen Kühr and van der Vlugt, 1934). This theory was thought to be disproven by studies showing that only microorganisms isolated with Fe(0) as sole electron donor were capable of increasing anoxic corrosion, while strains isolated with H_2_ as electron donor were not (Dinh et al., 2004; Mori et al., 2010; Uchiyama et al., 2010; Enning and Garrelfs, 2014; Kato et al., 2015). Those studies, however, did not consider that hydrogenotrophic microorganisms can differ strongly in their H_2_ consumption characteristics, as explained in this work for acetogens. Moreover, it is very likely that enrichments with Fe(0) as electron donor select for strains with a low H_2_ threshold and high H_2_ affinity, while isolations with H_2_ (often high H_2_ partial pressure) select for strains with a high growth yield and growth rate, but a high H_2_ threshold and low H_2_ affinity.

**FIGURE 6:**
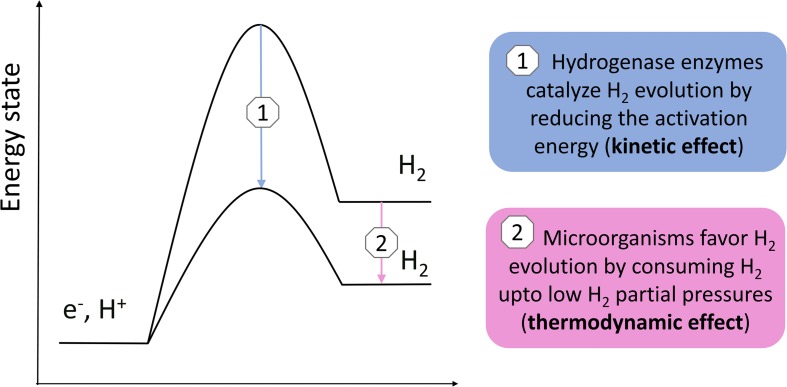
Simple scheme illustrating the two different mechanisms by which H_2_ consuming microorganisms possibly increase the H_2_ evolution reaction on a cathode or Fe(0).

Previous studies demonstrated that acetogens stimulate H_2_ evolution on a cathode and Fe(0) through the excretion of hydrogenases or other components catalyzing the H_2_ evolution reaction (Deutzmann et al., 2015; Philips et al., 2019; Tremblay et al., 2019). This work used the Butler–Volmer Equation (Equation 2) to demonstrate that the H_2_ evolution rate (current) on a cathode depends both on the exchange current density (kinetic effect, related to catalysis by enzymes) (Figure 3B) and the H_2_ partial pressure (thermodynamic effect) (Figure 3A). Consequently, there are two mechanisms by which hydrogenotrophic microorganisms could increase the H_2_ evolution rate on a cathode or Fe(0) (Figure 6): (1) catalysis of the H_2_ evolution reaction by extracellular hydrogenases or other components; and (2) the maintenance of low H_2_ partial pressures by H_2_ consumption. These two mechanisms are not mutually exclusive, but likely reinforce each other. The relative importance of each mechanism possibly depends on the reactivity of the material (higher for Fe(0) than for carbon-based cathodes), as well as on the H_2_ consumption characteristics and enzyme secretion mechanisms of the involved strains.

In addition, more EET mechanism than presented in Figure 1 could exist, while strains could combine different EET mechanisms or adjust their EET mechanism depending on the conditions (for instance cathode potential). Moreover, the presence of cytochromes in the acetogenic *Sporomusa* and *Moorella* spp. definitely warrants further investigation of a possible direct EET mechanism.

The focus here was solely on acetogenic bacteria, but also other hydrogenotrophic microorganisms, e.g., methanogens and sulfate reducers, could favor the H_2_ evolution reaction on a cathode or Fe(0) by maintaining low H_2_ partial pressures. Methanogens differ in their H_2_ threshold and H_2_ affinity (Thauer et al., 2008) and a correlation between their H_2_ threshold and Fe(0) corrosion rate was already suggested (Palacios Jaramillo, 2019). In addition, some methanogens were found to excrete hydrogenase enzymes to catalyze the H_2_ evolution reaction (Deutzmann et al., 2015; Tsurumaru et al., 2018), while evidence exist that some methanogenic strains have a direct EET mechanism (Beese-Vasbender et al., 2015a; Rowe et al., 2019; Yee et al., 2019). Also the sulfate reducing IS4 strain likely has a direct EET mechanism (Beese-Vasbender et al., 2015b). Consequently, different strategies to obtain extracellular electrons from solid electron donors probably occur in the microbial world (Figure 1).

Acetogens and other hydrogenotrophic microorganisms capable of using a solid electron donors are of interest for biotechnological applications (e.g., microbial electrosynthesis), while they could also cause microbial induced corrosion and impact biogeochemical cycles. A good understanding of the role of microorganisms in those processes requires fundamental insights into their EET mechanism. I hypothesize here that the EET mechanism of acetogenic bacteria depends on their H_2_ consumption characteristics. Hence, assessment of the H_2_ consumption characteristics of various acetogenic strains could be valuable to select the optimal strain for microbial electrosynthesis applications. Genetic engineering cannot change the H_2_ consumption characteristics as easy as it changes the resulting end-products (Humphreys and Minton, 2018), so target strains should be chosen based on their H_2_ consumption characteristics. In addition, a good understanding of strain related differences in the EET mechanism will improve the assessment of microbial influenced corrosion based on microbial community compositions.

## Conclusion

This work explained that the H_2_ partial pressure affects the H_2_ evolution reaction on a cathode or Fe(0) surface. This led to the assumption that the maintenance of low H_2_ partial pressures by hydrogenotrophic microorganisms is a mechanism to increase the H_2_ evolution reaction on a cathode or Fe(0), in addition to the catalysis by extracellular hydrogenases or other components (Figure 6). The H_2_ consumption characteristics of acetogenic bacteria were further discussed, which suggested that acetogens differ in their H_2_ threshold and H_2_ consumption kinetic parameters. Consequently, I hypothesize that the differences in the capacity of acetogens to use a solid electron donors, e.g., cathode and Fe(0), are related to the differences in their H_2_ consumption characteristics. The focus here was on acetogenic bacteria, but similar considerations are likely also relevant for other hydrogenotrophic microorganisms capable of using a cathode or Fe(0) as electron donor.

## Data Availability Statement

The raw data supporting the conclusions of this article will be made available by the authors, without undue reservation, to any qualified researcher.

## Author Contributions

The author confirms being the sole contributor of this work and has approved it for publication.

## Conflict of Interest

The authors declare that the research was conducted in the absence of any commercial or financial relationships that could be construed as a potential conflict of interest.
